# FDG-PET assessment of the locus coeruleus in Alzheimer’s disease

**DOI:** 10.1016/j.ynirp.2020.100002

**Published:** 2021-02-01

**Authors:** Kathy Y. Liu, Julio Acosta-Cabronero, Young T. Hong, Yeo-Jin Yi, Dorothea Hämmerer, Robert Howard, for the Alzheimer’s Disease Neuroimaging Initiative

**Affiliations:** a Division of Psychiatry, University College London, UK; b Wellcome Centre for Human Neuroimaging, UCL Institute of Neurology, University College London, UK; c Tenoke Ltd., Cambridge, UK; d Wolfson Brain Imaging Centre, University of Cambridge, UK; e German Center for Neurodegenerative Diseases (DZNE), Magdeburg, Germany; f Institute of Cognitive Neurology and Dementia Research, Otto-von-Guericke-University Magdeburg, Magdeburg, Germany; g Institute of Cognitive Neuroscience, University College London, UK

**Keywords:** Locus coeruleus, Alzheimer disease, Positron-emission tomography, Fluorodeoxyglucose F18

## Abstract

Sensitive and reliable *in vivo* imaging of the locus coeruleus (LC) is important to develop and evaluate its potential as a biomarker in neurodegenerative diseases such as Alzheimer’s disease (AD). It is not known whether AD-related alterations in LC integrity can be detected using ^18^F-labelled fluoro-2-deoxyglucose (FDG) positron emission tomography (PET). Mean FDG-PET images from AD patients (N ​= ​193) and controls (N ​= ​256) from the ADNI database were co-registered to a study-wise anatomical template. Regional LC median standardized uptake value ratio (SUVR) values were obtained using four previously published LC masks and normalized to values from pons and cerebellar vermis reference regions. To support the validity of our methods, other regions previously reported to be most and least affected metabolically in AD were also compared to controls. The LC did not show between-group differences in FDG-PET signal, whereas the mammillary bodies did, despite these regions having comparable volumes and SUVR ranges. Brain regions previously reported to be most and least affected metabolically in AD compared to controls showed medium-to-large and small effect sizes for SUVR differences respectively. The results do not support the current application of LC FDG-PET signal as an *in vivo* biomarker for AD. Methodological and demographic factors potentially contributing to these findings are discussed. Future research may investigate age-related differences in LC FDG-PET signal and higher resolution images to fully explore its biomarker potential.

## 1 Introduction

The locus coeruleus (LC), the major noradrenergic (NA) nucleus of the brain, regulates arousal, cognitive and autonomic functions (Samuels and Szabadi, 2008; Sara, 2009). In Alzheimer’s disease (AD), early neuropathological changes in this structure (Braak et al., 2011) precede substantial neuronal loss (Kelly et al., 2017; Theofilas et al., 2017; Zarow et al., 2003) and associated cognitive (Grudzien et al., 2007; Kelly et al., 2017) and neuropsychiatric (Ehrenberg et al., 2018; Oh et al., 2019) symptoms. Optimal *in vivo* imaging of the LC has been highlighted as an important goal to evaluate its potential to be a biomarker in neurodegenerative diseases (Betts et al., 2019b), and assess potential efficacy of treatments targeting the LC-NA system.

Previous studies, using T1 or magnetization transfer (MT)-weighted magnetic resonance imaging (MRI) approaches, reported lower LC MRI signal intensity (an *in vivo* measure of LC structural integrity reflecting cell density (Keren et al., 2015; 2009; Liu et al., 2018)) in patients with AD compared to controls (Betts et al., 2019a; Takahashi et al., 2015), consistent with post-mortem findings of LC neuron loss in AD (Braak et al., 2011; Kelly et al., 2017; Theofilas et al., 2017). Functional imaging of the LC *in vivo* is a challenge, owing to its small and elongated structure (~15 ​mm in length and 1–3 ​mm in diameter (Fernandes et al., 2012)) relative to the spatial resolution limits of functional MRI (fMRI) and positron emission tomography (PET) techniques, which are typically larger than 3 ​mm isotropic resolution, and accurate co-registration processes have been recommended to augment the signal-to-noise ratio (Betts et al., 2019b). Nonetheless, numerous fMRI studies have consistently shown LC activation by tasks or stimuli that induce stress or involve switching attention in response to stimuli change (Liu et al., 2017), consistent with models of LC function, and two previous ^18^F-labelled fluoro-2-deoxyglucose (FDG) PET studies (in healthy younger adults, N ​≤ ​25) reported diurnal differences in LC glucose metabolism (Buysse et al., 2004; Germain et al., 2007), but using a volume of interest (10mmx10mmx8mm) larger than the actual dimensions of the LC. The mammillary bodies, each with a comparable volume to the LC, showed AD-related FDG-PET signal differences (Nestor et al., 2003), demonstrating the potential high sensitivity of FDG-PET for small structures. It is not known whether AD-related alterations in LC integrity can be detected using FDG-PET.

PET with ^18^F-labelled fluoro-2-deoxyglucose (FDG) is widely believed to allow visualization and quantification of the resting state cerebral metabolic rate of glucose (CMRglc), and indirectly measure synaptic activity (Jueptner and Weiller, 1995), but it may also measure other or multiple biological processes, including impaired glucose blood brain barrier transport and vascular dysfunction in AD (Sweeney et al., 2019). Although the precise mechanism underlying the FDG-PET signal is not fully established, a consistent pattern of regional alterations in AD has been reported (Herholz, 2010; Meltzer et al., 1996; Mosconi, 2013; Mosconi et al., 2010), which can be utilized as a biomarker to stage the severity of AD (Jack et al., 2018; Ou et al., 2019). Earlier studies reported that impaired glucose metabolism, or reduced FDG-PET signal, in regions including temporoparietal and/or posterior cingulate cortical areas, reliably predicted rapid progression to dementia in mild cognitive impairment patients (Herholz, 2010; Ou et al., 2019). Given evidence that tau pathology, which affects the LC early in AD (Braak et al., 2011), underlies neurodegeneration, subsequent atrophy and reduced PET-FDG signal (hypometabolism) (La Joie et al., 2012), it would be important to determine whether reduced LC integrity can be detected using FDG-PET to investigate its potential as a biomarker in AD.

We hypothesized that the LC would show reduced FDG-PET signal compared to controls. We used imaging data from the Alzheimer’s Disease Neuroimaging Initiative (ADNI) database and previously published LC masks (Betts et al., 2017; Dahl et al., 2019; Keren et al., 2009; Liu et al., 2018) to investigate whether reduced LC integrity in AD could be detected using FDG-PET.

## 2 Materials and methods

### 2.1 Subjects

All data used in the preparation of this article were obtained from the ADNI database (adni.loni.usc.edu). ADNI was launched in 2003, with the primary goal of testing whether serial magnetic resonance imaging (MRI), positron emission tomography (PET), other biological dementia markers, and structured clinical and neuropsychological assessment could be combined to measure the progression of patients with mild cognitive impairment (MCI) and early Alzheimer’s disease (AD). Written informed consent was obtained from all participants and recruitment was approved by the Institutional Review Boards of all of the participating institutions. Full details of ethics approval, study design, participant recruitment, and clinical testing have been published previously and are available at adni-info.org. The initial five-year study (ADNI-1 phase) that started in 2004 was extended by two years in 2009 (ADNI-GO phase) and received further funding for extension in 2011 and 2016 (ADNI-2, and ADNI-3 phases, respectively). New participants were recruited across North America during each phase of the study.

We included all cognitively healthy controls (HC) and patients clinically diagnosed with AD from ADNI Phases 1, 2 or 3 who had both mean co-registered FDG-PET and T1-weighted structural MR images on the ADNI database (AD N ​= ​242, HC N ​= ​329, total N ​= ​571). As many subjects had repeat FDG-PET and/or MRI imaging over several years, the earliest single FDG-PET and structural MRI images with the shortest intervening gap between them were used in the analysis. This was based upon the assumption that the LC would show detectable structural impairment by the time an individual had been diagnosed with AD, and concern that more advanced AD would be accompanied by greater generalized structural brain changes, that could affect the accuracy of image co-registration. All but 27 of the 571 subjects (>95%) had FDG-PET and structural MRI images that were dated less than one year apart.

### 2.2 Imaging protocol

All subjects were scanned using a standardized MRI protocol developed for ADNI (Jack et al., 2008). T1-weighted, three-dimensional (3D) magnetization-prepared rapid gradient-echo (MPRAGE) sequences were obtained using either a 1.5T or 3T MRI scanner in ADNI Phase 1 and a 3T scanner in ADNI Phases 2 and 3 (see Table 1 for participant numbers). Representative imaging parameters were as follows: repetition time ​= ​2,300 ​ms; inversion time ​= ​1,000 ​ms; flip angle ​= ​8°; field of view ​= ​240 ​× ​240 ​mm; and acquisition matrix of 192 x192 (256 x 256 for 3 ​T) ​× ​166 ​ ​mm yielding a voxel resolution of approximately 1 x 1 ​× ​1.2 ​mm.

**Table 1:** Number of participants from AD and HC groups recruited to each ADNI phase and scanned using 1.5T or 3T MRI scanners, who were included in the analysis.

Group	ADNI Phase (N)			Scanner strength	
	1	2	3	1.5T	3T
**AD**	63	118	12	63	130
**HC**	105	148	3	105	151

For all subjects, a dynamic 3D PET scan was conducted, in which six 5-min frames were acquired 30–60 ​min post injection with a dose of 185 MBq (ADNI 1 and 2) or 185 MBq ​±10% (ADNI 3) of ^18^F-FDG in a resting state with eyes open in a quiet darkened room (see Supplemental Table 1 for scanner types used at individual ADNI sites). Raw PET images from all sites were downloaded for quality control at the University of Michigan where separate frames were co-registered to the first extracted frame of the raw image file and then averaged to create a single 30 ​min PET image set in native space. Raw PET data were finally converted to DICOM file format and uploaded to the ADNI database.

### 2.3 Image co-registration and analysis

**Fig. 1:**
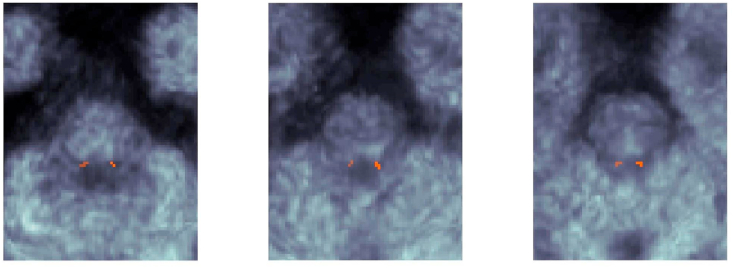
Example of the study co-registration accuracy protocol, which shows an individual’s co-registered FDG-PET images (at 3 different axial slices) and corresponding LC mask (Betts) position.

**Fig. 2:**
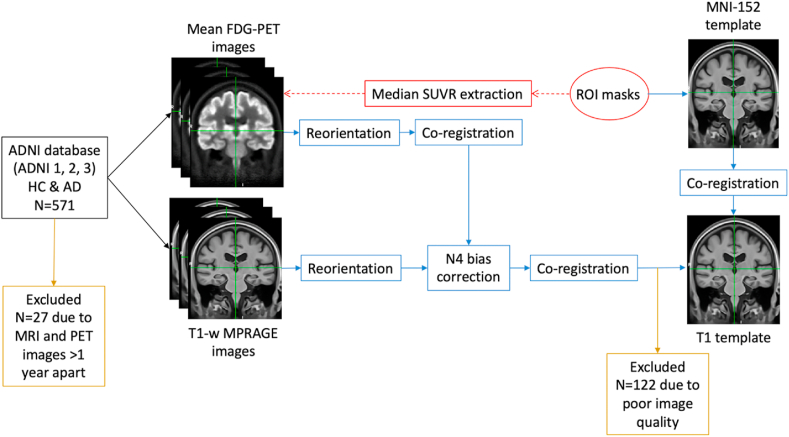
**Flow diagram representing the analysis pipeline.** After co-registration of 1) individual mean FDG-PET images to their corresponding N4 bias corrected MPRAGE image, 2) individual MPRAGE images to a study-wise T1 template, and 3) ROI space-defining (MNI-152) template to the study-wise T1 template, ROI masks were spatially transformed to each PET image in a single step to extract median SUVR values, which were normalized to cerebellar vermis or pons.

Mean FDG-PET and MPRAGE images were downloaded from the ADNI database in NIfTI format and reoriented to standard radiological orientation using the fslreorient2std tool in FSL. Each subject’s MPRAGE image dataset was corrected using a N4 bias field correction procedure (Avants et al., 2011; Tustison et al., 2010) and spatially co-registered to a common coordinate system using an iterative template generation routine (multi-resolution using mutual information as the cost metric for rigid and affine steps and cross-correlation for non-linear co-registration) available from ANTS v2.1 software package (http://stnava. github.io/ANTs). At this stage, we excluded 122 datasets (resulting in AD N ​= ​193, HC N ​= ​256, total N ​= ​449, see Table 1) due to poor structural MRI data quality (visual rating carried out by KL and JA-C). Individual mean FDG-PET images were then co-registered to the corresponding N4 bias corrected MPRAGE images using a two-stage (rigid then non-linear) co-registration routine in ANTS v2.1 (multi-resolution using mutual information as the cost metric throughout). To check for co-registration accuracy, all co-registered FDG-PET images were visually inspected for gross alignment in the brainstem region (see Supplemental Videos 1 and 2) and for the precision of LC mask location (Fig. 1). The analysis pipeline is represented as a flow diagram in Fig. 2.

A semi-quantitative measure of the FDG-PET signal is the region of interest (ROI) to reference region standard uptake value ratio (SUVR), which allows visualization and localization of what are widely presumed to be metabolic differences (Mosconi, 2013). Regional median standard uptake values (SUV) for each subject were obtained by spatially transforming region of interest (ROI) masks from their respective template space to individual native PET image spaces as follows: the ROI space-defining template, i.e. MNI152 (Montreal Neurological Institute, McGill University, Canada), was co-registered to our study-wise template using an ANTS based multi-resolution approach (mutual information and cross-correlation as the cost metrics for rigid/affine and non-linear steps, respectively). Subsequently, a single step (applying the global composition of pre-calculated transformations and nearest neighbor interpolation) spatially transformed each ROI mask to each PET image space.

We defined the LC ROI using four previously published individual LC masks (Betts et al., 2017; Dahl et al., 2019; Keren et al., 2009; Liu et al., 2018). The Betts and Liu LC masks were manually segmented from T1 or MT-weighted MRI template images from healthy adults, and Keren and Dahl LC masks were binarized LC probability maps that represented the spatial distribution of LC maximum signal intensity (at 1SD from the mean for Keren LC mask). To support the validity of our methods, we also examined regions previously reported to be most affected metabolically in AD compared to healthy controls with PET, including posterior cingulate cortex (PCC), anterior cingulate cortex (ACC), bilateral amygdala and mammillary bodies (Nestor et al., 2003), as well as regions where metabolism has been reported to be relatively preserved (Herholz et al., 1993), including primary visual cortex, putamen, pre and postcentral gyrus (primary sensorimotor cortex), cerebellum, thalamus and pons (Kalpouzos et al., 2009; Minoshima et al., 1995) (although significant hypometabolism has been demonstrated in the anterior thalamic nucleus (Nestor et al., 2003), believed to be part of a limbic-diencephalic network (Acosta-Cabronero and Nestor, 2014; Aggleton et al., 2016)). The mammillary body ROI was of particular interest to us because it is comparable in volume to the LC (around 100 ​mm^3^ (Kumar et al., 2008) and 70 ​mm^3^ (Fernandes et al., 2012) respectively). These non-LC bilateral ROIs were binary masks created using probability maps from standardized atlases (apart from the pons ROI, which was obtained from a discrete standardized atlas) in MNI52 space available from FSLeyes (https://fsl.fmrib.ox.ac.uk/fsl/fslwiki/FSLeyes), thresholded at 50% and visually inspected to determine accurate coverage of the relevant brain region. Specifically, the V1 visual cortex and mammillary body ROIs originated from the Juelich Histological atlas; the pons was defined by the pontine crossing tract ROI from the JHU DTI-based white-matter atlas, the cerebellar vermis was the combination of vermis VI, VIIb, VIIIa/b, IV, X, Crus I and Crus II ROIs from the cerebellar atlas in MNI-152 space; and the remaining ROIs were taken from the Harvard-Oxford cortical or subcortical atlases. Details of the atlases included in FSL can be found here: https://fsl.fmrib.ox.ac.uk/fsl/fslwiki/Atlases.

### 2.4 Statistical analysis

The SUVR method calculates the ratio of voxel intensity to a reference region intensity to correct for nonspecific radiotracer uptake and enables semi-quantitative comparisons between different scans and subjects. However, as non-AD conditions such as microcerebral vascular disease may affect reference region metabolism in older adults (DeCarli et al., 1996), selection of the most appropriate region has been debated. Hence, we normalized median ROI SUV values to both the cerebellar vermis and pons, as these have been the most widely used reference regions in past FDG-PET studies in Alzheimer’s disease (Meltzer et al., 1996; Minoshima et al., 1995; Nestor et al., 2003; Scheltens et al., 2018). Differences in regional mean SUVR values between AD and HC groups were assessed using unpaired Student t-tests with an alpha level of 0.05 and Cohen’s effect size *d* (standardized d ​= ​0.2 considered ‘small’, d ​= ​0.5 ‘medium’ and d ​= ​0.8 ‘large effect size) (Cohen, 2013), except when groups had unequal variances, where Welch’s t-tests and Cohen’s d with assumption of non-homogeneity of variance were used.

### 2.5 Post hoc analyses

To account for the low spatial resolution of PET scans (with respect to typical MRI resolution), Betts, Keren and Dahl LC masks (all significantly smaller than the Liu LC mask), were dilated using a spherical kernel of 1 ​mm radius, and the Dahl mask was dilated a second time using a 1.5 ​mm radius kernel because it represented only the spatial distribution of the peak LC signal, leading to four additional (larger) Betts, Keren and Dahl (1 ​mm) and Dahl (1.5 ​mm) LC masks respectively. To also account for the possibility that the LC is smaller in AD compared to HCs, resulting in a higher proportion of lower intensity voxels due to atrophy alone, we extracted the maximum (98th percentile) SUVR values from the LC and mammillary bodies ROIs, as both are relatively small ROIs and known to undergo significant cell loss in AD (Copenhaver et al., 2006; Kelly et al., 2017).

## 3 Results

The AD group had significantly fewer years of education and lower MMSE scores, but there were no other significant differences in demographic factors (Table 2).

**Table 2:** Demographic features in Alzheimer’s disease and healthy control groups.

Demographic variable	AD (N ​= ​193)	HC (N ​= ​256)	Differences between groups
Age (mean years [SD])	74.6 [8.0]	74.5 [5.8]	∗t(326) ​= ​0.073, p ​= ​0.942
Female (%)	77 (40)	122 (48)	X2(1) ​= ​2.685, p ​= ​ 0.101
Education (mean years [SD])	15.5 [3.0]	16.3 [2.8]	**t(443)** ​= ​**-2.963, p** ​= ​**0.003**
MMSE (mean score [SD])	23 [2.1]	29 [1.1]	**∗t(270) ​= ​-34.65, p ​< ​2.2e-16**

Abbreviations: AD - Alzheimer’s disease, HC - healthy control, MMSE - Mini-Mental State Examination, SD ​= ​standard deviation. Chi-squared (X2) test was used to assess the difference in sex between groups. For other demographic variables, variances between groups were assessed using F-test of equality of variances. Differences between groups were calculated using either Student t-tests for equal variances or Welch’s *t*-test (∗) for unequal variances. In the AD group, there was missing data for N ​= ​4 subjects in all demographic categories except for Female (%). Significant differences are highlighted in bold.

**Fig. 3:**
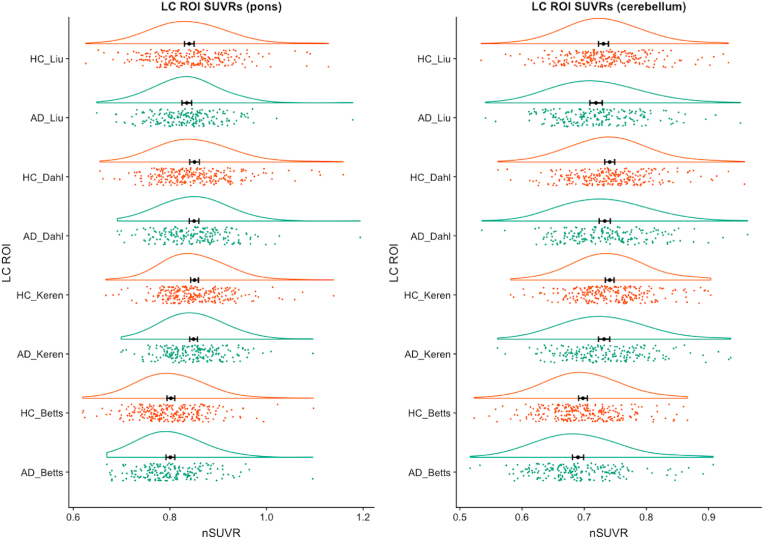
**Raincloud plots showing regional SUVR values for the four original (non-dilated) LC masks (Liu, Dahl, Keren, Betts) in AD and HC groups, using either the pons (left) or cerebellar vermis (right) as reference regions.** Abbreviations: HC - healthy control, AD - Alzheimer’s disease, ROI - region of interest, SUVR - normalized mean standardized uptake ratio. The black dot and vertical lines indicate mean and 95% confidence intervals (for the mean), the green and orange data points represent individual SUVR values for AD and HC groups respectively, and the curved lines represent density plots.

Compared to previously published ex vivo dimensions (14.5 ​mm ​× ​2 ​mm×2 ​mm per LC and an estimated total volume of around 116 ​mm^3^) (Fernandes et al., 2012), the volumes of Betts, Keren, Liu, Dahl LC masks in the study-wise space were 89 ​mm^3^ (98 voxels), 78 ​mm^3^ (86 voxels), 195 ​mm^3^ (216 voxels) and 19 ​mm^3^ (21 voxels) respectively, and the *dilated* Betts, Keren, Dahl (1 ​mm) and Dahl (1.5 ​mm) mask volumes were 214 ​mm^3^ (237 voxels), 201 ​mm^3^ (222 voxels), 64 ​mm^3^ (71 voxels) and 212 ​mm^3^ (235 voxels) respectively. There were no significant differences in LC SUVR (median or maximum) values between AD and HC groups using any of the four original LC masks or dilated Betts, Keren and Dahl LC masks, and all effect sizes were negligible (Cohen’s d ​< ​0.2) (Fig. 3 and Supplemental Table 2). This remained the case after adjusting for years of education, by including this as an additional covariate in a one-way ANCOVA.

**Fig. 4:**
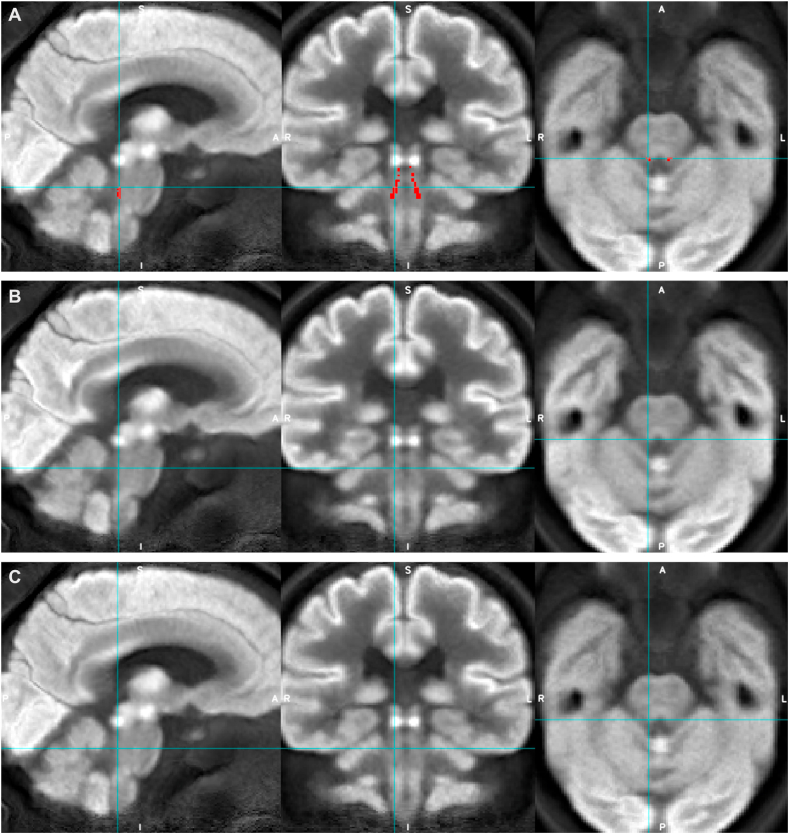
Appearances of the average group FDG-PET image for AD (rows A and C) and HC (row B) groups with Betts LC mask overlaid (red, row A, and blue crosshairs) to demonstrate LC ROI location.

**Fig. 5:**
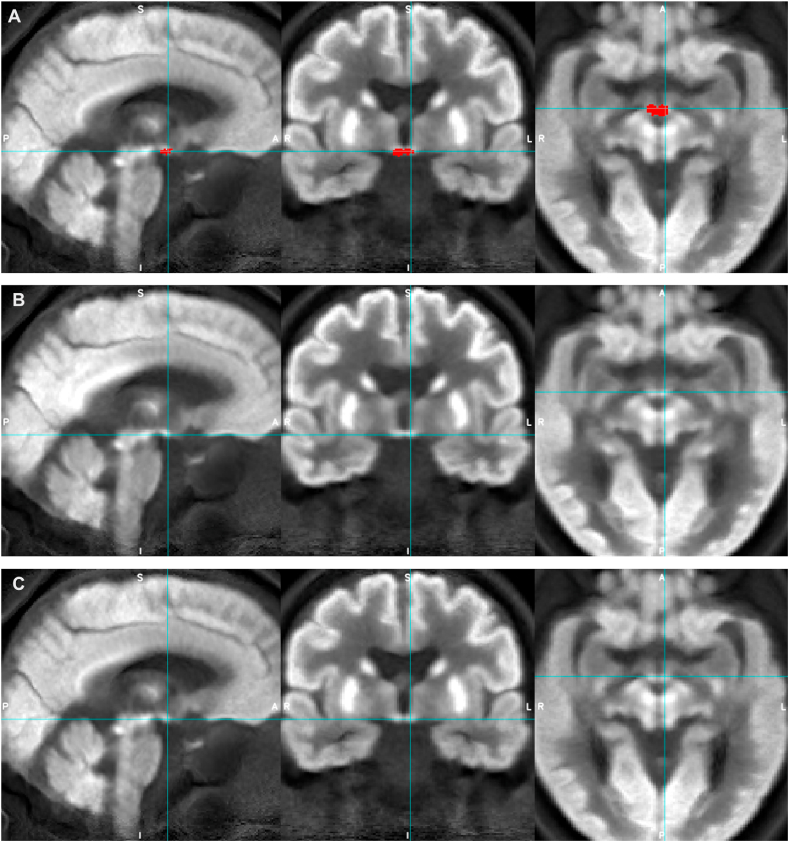
Appearances of the average group FDG-PET image for AD (rows A and C) and HC (row B) groups with mammillary bodies mask overlaid (red, row A, and blue crosshairs) to demonstrate mammillary bodies ROI location and its relative visibility compared to the LC.

Although the non-dilated LC ROIs (apart from Dahl LC ROI) were comparable to the bilateral mammillary body ROI both in terms of volume (between 78 and 195 ​mm^3^ and 130 ​mm^3^ respectively) and SUVR range (0.69–0.85 and 0.64–0.79 respectively), only the mammillary bodies showed significant group differences in median and maximum SUVR, with medium effect sizes (d ​= ​0.5–0.6) and could be visualized on the average group PET images more clearly (Fig. 4, Fig. 5).

**Fig. 6:**
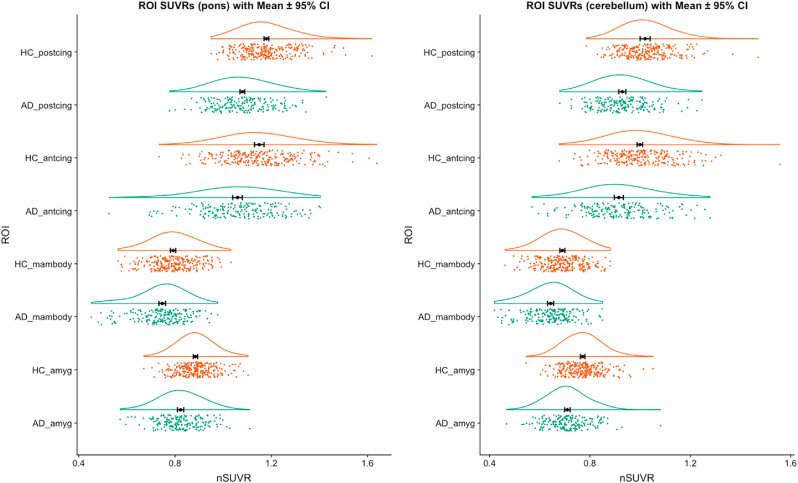
**Raincloud plots showing regional SUVR values for ROIs most expected to show reduced metabolism in AD compared to HC groups, using either the pons (left) or cerebellar vermis (right) as reference regions.** Abbreviations: HC - healthy control, AD - Alzheimer’s disease, postcing - posterior cingulate, antcing - anterior cingulate, mambody - mammillary bodies, amyg - bilateral amygdala, ROI - region of interest, SUVR - normalized mean standardized uptake ratio. The black dot and vertical lines indicate mean and 95% confidence intervals (for the mean), the green and orange data points represent individual SUVR values for AD and HC groups respectively, and the curved lines represent density plots.

In addition to the mammillary bodies, all other regions anticipated to have lower metabolism in AD versus controls (PCC, ACC, bilateral amygdala and mammillary bodies), showed significant reductions in SUVR with medium to large effect sizes (d ​= ​0.5–0.8) (Fig. 6 and Supplemental Table 2). Regions anticipated to have relatively preserved metabolism mostly showed small reductions in AD versus controls (d ​= ​0.2–0.5), including pre and postcentral gyri, thalamus and primary visual cortex. Differences in the putamen were only detected when the cerebellar vermis and not pons was used as the reference region. There were no between-group SUVR differences in the pons or cerebellar vermis when normalized to vermis and pons respectively (Supplemental Table 2 and Supplemental Fig. 1).

## 4 Discussion

Using a large sample of co-registered mean FDG-PET images (N ​= ​449) from the ADNI database, this study investigated LC FDG-PET signal differences between AD and healthy control groups. We found medium-to-large and small effect sizes for differences in SUVR from brain regions previously reported to be most and least affected metabolically in AD compared to healthy controls respectively, broadly supporting the validity of our co-registration and analysis methods. However, the LC did not show between-group differences in FDG-PET signal, whereas the mammillary bodies did, despite these regions having comparable volumes and SUVR ranges. This suggests that the volume of the LC mask, the methods used to calculate SUVR and the signal intensity range in the LC were unlikely to be reasons why SUVR differences were not detected in the LC. We discuss other potential factors that may have resulted in this negative finding below.

Although we had hypothesized that individuals with AD would show impaired LC function secondary to cell loss versus controls, alternative alterations affecting the LC-NA system may have contributed to our findings. For example, there may have been increased compensatory activity of the surviving LC neuronal population secondary to cell loss in AD, which was reported in a previous study (Hoogendijk et al., 1999), and/or age-related loss of LC structural integrity in the healthy older adult group (Betts et al., 2017; Clewett et al., 2016; Liu et al., 2018; Manaye et al., 1995; Mann and Yates, 1974; Shibata et al., 2006), which may have reduced the signal-to-noise ratio and limited the ability to detect differences between the groups. As age-related FDG-PET signal changes affect several brain regions (Mosconi, 2013), possibly including the LC, it could be informative to obtain and compare LC FDG-PET data between healthy older and younger adults.

The ability to detect *in vivo* LC differences in AD versus older control groups using FDG-PET may have been limited by methodological constraints. For example, as the LC is an elongated but not perfectly cylindrical structure (approximately 14.5 ​ ​mm ​× ​2.5 ​ ​mm x 2 ​mm) and tilted in plane in three dimensions, it is potentially more susceptible to partial volume effects due to the relatively large voxel dimensions (≥3 ​mm^3^ isotropic) of ADNI FDG-PET scans, compared to the spherical mammillary bodies (5 ​mm in diameter), especially if there was relatively low signal contrast between LC compared to surrounding brainstem tissue. One suggested strategy to mitigate partial volume effects at the boundary of the structure has been to assess single, peak voxels (Keren et al., 2009; Liu et al., 2017), but we did not find any group differences using the Dahl LC mask, which represented a distribution of LC peak MRI signal intensity.

We used SUVR, which is not linearly related to quantitative measures of CMRglc and relies on the measurement stability of a reference region, thus it is potentially less reliable for calculating the magnitude of metabolic differences between groups. However, neither the pons nor cerebellar vermis reference regions in the study showed SUVR differences between AD and control groups. A quantitative method in a highly controlled environment (e.g. a dynamic modelling approach to calculate binding potentials with partial volume correction and use of the same scanner for all subjects), may have provided additional power to detect differences in small structures such as the LC. However, when developing optimal *in vivo* imaging methods in older patients with dementia, it is relevant to consider that compared to quantitative methods, SUVR is associated with a more tolerable PET procedure with reduced scanning time and no need for blood sampling. Given the discussion in the literature on the underlying mechanism of the FDG-PET signal, we did not aim to investigate the metabolic state of the LC in AD versus controls, but primarily investigated LC FDG-PET signal as a potential biomarker in AD.

We tried to optimize the precision of our LC measurements using four previously published LC masks as our LC ROIs instead of a larger, less specific volume of interest, as was reported in previous studies, relative to the reported ex vivo dimensions of the LC. We also strived to optimize co-registration precision by using rigid and affine nonlinear co-registration and visually checking its accuracy and tried to account for low image resolution and potential atrophy by additionally employing mask dilation and extracting the maximum (98th percentile) SUVR values. Although it is possible that alternative co-registration or signal extraction techniques may have offered even further precision, it is unclear how much benefit this would have offered in the context of pre-existing functional images with limited resolutions. It would be interesting for future studies to explore whether higher resolution images, if available, could provide alternative findings.

In conclusion, our findings do not support the current application of LC FDG-PET signal as a potential *in vivo* biomarker in AD, as there were no detectable differences between AD patients and controls in our sample. It would be informative to investigate age-related differences and/or longitudinal differences with AD progression to fully explore its biomarker potential.

## Declaration of competing interest

The authors declare the following financial interests/personal relationships which may be considered as potential competing interests: JAC has equity and a full-time appointment at Tenoke Limited, which provides medical imaging services. All other authors report no conflict of interest.
