# Supplementary material for: FDG-PET assessment of the locus coeruleus in Alzheimer’s disease
Source: Neuroimage Rep. 2021 Jan 25;1(1):100002. doi: 10.1016/j.ynirp.2020.100002 (PMC8262255; doi:10.1016/j.ynirp.2020.100002)

SUPPLEMENTAL DATA

**Supplemental Table 1: Overview of PET scanner types and included subjects from ADNI database.**

| **PET scanner Manufacturer** | **Type** | **Number of subjects** | |
| --- | --- | --- | --- |
|  |  | **AD** | **HC** |
| Philips Medical Systems | (Not reported) | 2 | 5 |
|  | Allegro Body | 2 | 3 |
|  | G-PET Brain | 2 | 3 |
|  | GEMINI- TF | 6 | 15 |
|  | Guardian body | 2 | 5 |
|  | Ingenuity | 2 | 0 |
| Siemens | 1093 | 2 | 7 |
|  | HR+ | 52 | 60 |
|  | 1094 | 10 | 15 |
|  | Accel | 3 | 6 |
|  | ECAT HRRT | 17 | 24 |
|  | Biograph 64 | 7 | 3 |
|  | Biograph 128 | 1 | 0 |
|  | Biograph 40 | 1 | 0 |
|  | LSO PET/CT | 15 | 24 |
|  | SOMATOM | 3 | 0 |
|  | Exact | 0 | 2 |
| GE Medical Systems | Advance | 12 | 12 |
|  | Discovery 600 | 4 | 1 |
|  | Discovery LS | 13 | 15 |
|  | Discovery HR | 1 | 0 |
|  | Discovery MI | 3 | 0 |
|  | Discovery RX | 3 | 4 |
|  | Discovery ST/STE | 31 | 52 |

**Supplemental Videos 1 and 2: Video of individual co-registered FDG-PET images in the AD (1) and CN (2) group created to visually check for gross co-registration accuracy.**

1. [**Click here to watch video**](https://tinyurl.com/y6gwgrhj)
2. [**Click here to watch video**](https://tinyurl.com/y5lgevsz)

**Supplemental Table 2: Differences in regional SUVR values between AD and HC groups using either pons or cerebellar vermis as reference regions.** Abbreviations: HC - healthy control, AD - Alzheimer’s disease, ROI - region of interest, SUVR - normalized mean standardized uptake ratio, PCC - posterior cingulate cortex, ACC - anterior cingulate cortex. Differences between groups and effect sizes were calculated using Student t-tests and Cohen’s d, except for groups with unequal variances (*), which used Welch’s t-tests and a Cohen’s d variant assuming non-homogeneity of variance. Variances in SUVR ROIs between groups were assessed using F-test of equality of variances. Statistically significant differences (p<0.05) are highlighted in bold.

| ROI | Mean SUVR  (pons, cerebellum) | | Differences between groups | |
| --- | --- | --- | --- | --- |
|  | AD | HC |  |  |
|  |  |  | Pons | Cerebellum |
| LC Betts | 0.801,  0.690 | 0.802,  0.698 | t(447)=-0.148, p=0.883  d=-0.014 (-0.201-0.173) | t(447)=-1.345, p=0.179  d=-0.128 (-0.316-0.059) |
| LC Keren | 0.849,  0.732 | 0.851,  0.741 | t(447)=-0.401, p=0.689  d=-0.038 (-0.226-0.149) | t(447)=-1.652, p=0.099  d=-0.158 (-0.345-0.030) |
| LC Dahl | 0.850,  0.733 | 0.851,  0.741 | t(447)=-0.139, p=0.889  d=-0.013 (-0.201-0.174) | t(447)=-1.371, p=0.171  d=-0.131 (-0.318-0.057) |
| LC Liu | 0.835,  0.719 | 0.840,  0.731 | t(447)=-0.773, p=0.44  d=-0.074 (-0.261-0.114) | t(447)=-1.968, p=0.05  d=-0.188 (-0.375-0.0001) |
| LC Betts (dilated) | 0.689,  0.689 | 0.698,  0.698 | t(447)=-1.611, p= 0.108  d=-0.154 (-0.341-0.034) | t(447)=-1.611, p=0.108  d=-0.154 (-0.341-0.034) |
| LC Keren (dilated) | 0.848,  0.731 | 0.852,  0.742 | t(447)=-0.648, p=0.517  d=-0.062 (-0.249-0.126) | t(447)=-1.882, p=0.060  d=-0.179 -(0.367-0.008) |
| LC Dahl (dilated 1mm) | 0.847,  0.730 | 0.849,  0.739 | t(447)=-0.328, p=0.743  d=-0.031 (-0.219-0.156) | t(447)=-1.566, p=0.118  d=-0.149 (-0.337-0.038) |
| LC Dahl (dilated 1.5mm) | 0.843,  0.727 | 0.847,  0.738 | t(447)=-0.638, p=0.524  d=-0.061 (-0.248-0.127) | t(447)=-1.881, p=0.061  d=-0.179 (-0.367-0.008) |
| LC Betts (max) | 0.822,  0.639 | 0.819,  0.643 | *t(440)=0.461, p=0.645  d=0.043 (-0.144-0.230) | t(447)=-0.608, p=0.544  d=-0.058 (-0.245-0.129) |
| LC Keren (max) | 0.871,  0.677 | 0.863,  0.677 | *t(439)=1.186, p=0.236  d=0.111 (-0.077-0.298) | t(447)=-0.038, p=0.970  d=-0.004 (-0.191-0.184) |
| LC Dahl (max) | 0.830,  0.646 | 0.821,  0.645 | *t(438)=1.353, p=0.177  d=0.126 (-0.061-0.314) | t(447)=0.152, p=0.879  d=0.014 (-0.173-0.202) |
| LC Liu (max) | 0.869,  0.676 | 0.863,  0.678 | t(447)=0.939, p=0.348  d=0.090, (-0.098-0.277) | t(447)=-0.273, p=0.785  d=-0.026 (-0.213-0.161) |
| PCC | 1.079,  0.929 | 1.177,  1.025 | **t(447)=-9.572, p<2.2e-16**  **d=-0.913 (-1.109- -0.716)** | **t(447)=-10.408, p< 2.2e-16**  **d=-0.992 (-1.191- -0.794)** |
| ACC | 1.063,  0.915 | 1.148,  0.999 | **t(447)=-6.071, p=2.72e-09**  **d=-0.579 (-0.770- -0.388)** | **t(447)=-7.052, p=6.749e-12**  **d=-0.672 (-0.865- -0.480)** |
| Amygdala | 0.823,  0.710 | 0.885,  0.771 | ***t(375)=-8.019, p=1.372e-14**  **d=-0.782 (-0.976- -0.588)*** | **t(447)=-8.472, p=3.521e-16**  **d=-0.808 (-1.002- -0.613)** |
| Mammillary bodies | 0.747,  0.643 | 0.792,  0.690 | **t(447)=-5.115, p=4.656e-07**  **d=-0.488 (-0.678- -0.298)** | **t(447)=-6.064, p=2.826e-09**  **d=-0.578 (-0.769- -0.387)** |
| Mammillary bodies (max) | 0.803  0.624 | 0.862  0.677 | **t(447)=-6.045, p=3.154e-09**  **d=-0.576 (-0.767- -0.385)** | **t(447)=-6.251, p=9.558e-10**  **d=-0.596 (-0.787- -0.404)** |
| Postcentral gyrus | 1.240,  1.067 | 1.276,  1.109 | **t(447)=-2.742, p=0.006**  **d=-0.261 (-0.449- -0.073)** | **t(447)=-4.217, p=3e-05**  **d=-0.402 (-0.591- -0.213)** |
| Precentral gyrus | 1.286,  1.107 | 1.321,  1.148 | ***t(441)=-2.8218, p=0.005**  **d=-0.263 (-0.451- -0.075)*** | **t(447)=-4.377, p=1.502e-05**  **d=-0.417 (-0.607- -0.228)** |
| Thalamus | 1.203,  1.036 | 1.244,  1.083 | **t(447)=-4.288, p=2.209e-05**  **d=-0.409 (-0.598- -0.220)** | **t(447)=-5.338, p=1.495e-07**  **d=-0.509 (-0.699- -0.319)** |
| Visual cortex | 1.364,  1.172 | 1.422,  1.235 | **t(447)=-3.540, p=4.414e-04**  **d=-0.338 (-0.526- -0.149)** | **t(447)=-5.568, p=4.459e-08**  **d=-0.531 (-0.721- -0.340)** |
| Putamen | 1.467,  1.264 | 1.491,  1.299 | t(447)=-1.799, p=0.073  d=-0.172 (-0.360- 0.016) | **t(447)=-2.810, p=0.005**  **d=-0.268 (-0.456- -0.079)** |
| Pons | 0.864 | 0.874 | - | t(447)=-1.335, p=0.183  d=-0.127 (-0.315-0.060) |
| Cerebellar vermis | 1.166 | 1.154 | t(447)=1.197, p=0.232  d=0.114 (-0.073- 0.302) | - |

**Supplemental Figure 1. Raincloud plots showing regional SUVR values for ROIs least expected to show reduced metabolism in AD compared to HC groups, using either the pons (left) or cerebellar vermis (right) as reference regions.** Abbreviations: HC - healthy control, AD - Alzheimer’s disease, cereb - cerebellar vermis, pons - pontine reference region, puta - putamen, thalam - thalamus, occ - primary visual (V1) cortex, precen - precentral gyrus, postcen - postcentral gyrus, ROI - region of interest, SUVR - normalized mean standardized uptake ratio. The black dot and vertical lines indicate mean and 95% confidence intervals (for the mean), the green and orange data points represent individual SUVR values for AD and HC groups respectively, and the curved lines represent density plots.


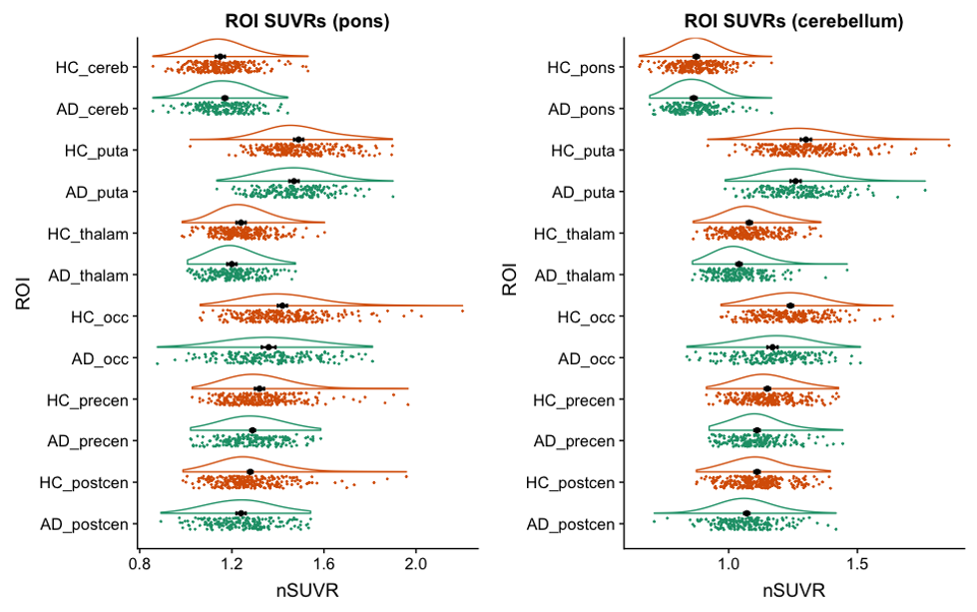

Supplement: Supplementary file 1 [file mmc1.docx]
